# Mechanical Properties of Bio-Based Sandwich Composites Containing Recycled Polymer Textiles

**DOI:** 10.3390/polym15183815

**Published:** 2023-09-19

**Authors:** Pooria Khalili, Mikael Skrifvars, Hom Nath Dhakal, Saeid Hosseinpour Dashatan, Mikael Danielsson, Alèxia Feiner Gràcia

**Affiliations:** 1 Swedish Centre for Resource Recovery, Faculty of Textiles, Engineering and Business, University of Borås, 510 90 Borås, Sweden; mikael.skrifvars@hb.se; 2 Advanced Polymers and Composites (APC), School of Mechanical Design and Engineering, University of Portsmouth, Portsmouth PO1 3DJ, UK; hom.dhakal@port.ac.uk; 3 Brunel Composite Centre, Brunel University London, London UB8 3PH, UK; saeid.dashatan@brunel.ac.uk; 4 Albany International AB, 302 41 Halmstad, Sweden; mikael.danielsson@albint.com; 5 Department of Textile Technology and Design, Universitat Politècnica de Catalunya-Barcelona Tech—UPC, 08034 Barcelona, Spain

**Keywords:** mechanical properties, sandwich composites, regenerated cellulose, impact behaviour

## Abstract

In this paper, sandwich composites were produced by compression moulding techniques, and they consisted of regenerated cellulose fabric (rayon) and bio-based polypropylene (PP) to form facings, while virgin and recycled polyamide (PA) textiles were used as core materials. To compare the mechanical performance between sandwich composites and typical composite designs, a control composite was produced to deliver the same weight and fiber mass fraction from rayon and PP. To evaluate the influence of recycled textile on the mechanical properties of the composites, a series of flexural, low velocity impact (LVI) and tensile tests were performed. It was found that the incorporation of thicker PA textile enhanced the bending stiffness by two times and the peak flexural force by 70% as compared to those of control. Substitution of a layer of recycled textile for two layers of rayon provided a good level of impact energy absorption capacity (~28 J) and maximum force (~4893–5229 N). The tensile strength of the four sandwich composites was reported to be in the range of 34.20 MPa and 46.80 MPa. This value was 91.90 for the control composite. The 2D cross-section slices of the composite specimens did not show any evidence of fiber tow debonding, fiber bundle splitting, or delamination.

## 1. Introduction

Demand for the utilisation of textile materials has been increasing in different industries, which leads to the accumulation of textile waste. While the rate of recycling is globally lesser than 20%, the rough estimation of the generation of textile waste is higher than 150 million tonnes [1,2]. A large quantity of these wastes is either discarded or incinerated, and this does not have a positive impact on the environment. When these large quantities of textiles are discarded in landfills as waste, they contaminate groundwater through decomposition and form micro-plastics and greenhouse gases [3]. The ever-growing world population, which brings increased consumption, has resulted in an acute shortage of resources [4,5]. Moreover, the adverse effects of synthetic waste materials are vast, ascribed to their toxicity and non-biodegradability. These factors have directly affected the textile industry and demonstrate the importance of recycling textile waste [6].

An enormous quantity of textile waste is generated in the paper, pulp, and textile industries throughout the production process. Rejected fabrics, fabric scraps, cutting waste, yarns, threads, and short fibers are major wastes in varying sections [7]. These textiles are greatly engineered textile structures composed of yarns of polyamides and polyester and are wove into various types of textile structures. Rather than disposing of textile materials and incinerating them after their life span, cleaning processes and various treatments can be performed on the textiles in order to utilise them as reinforcements for specific composite applications.

Use of recycled textiles contributes to reducing the creation of new fibrous materials through agricultural approaches and extraction, which lead to a higher carbon footprint. By valorizing the recycled textiles, there is a reduction in negative environmental impacts caused by discarding waste in the form of textiles [3].

Owing to damage, wear, and other issues, textile waste produced nowadays cannot be reused in production lines for consumer products. However, post-consumer waste possesses the potential to be utilised for diverse purposes, where its properties and performance can be utilised and enhanced.

In the paper machine, textile materials are called paper machine clothing and are used for the formation and dewatering of the pulp. The life span of these textiles is typically from 30 days to 90 days, depending on the position in the machine and the paper grade. After a full life cycle, the machine’s clothing must be replaced. There is therefore an interest in studying various ways to recycle or reuse these textiles as secondary products. The textile still retains good properties, such as strength and durability, which could be used in composite structures. Manufacturing semi-structural composite materials using textile waste is one of the most useful recycling methods that is sustainable.

Man-made regenerated fibers have been revealed to offer significant promise as reinforcements in thermoplastics for continuous [8] and short fiber systems [9,10]. They provide CO_2_ neutrality, low density, biodegradability, and non-abrasiveness to manufacturing equipment, the same as natural fibers (NFs) [11,12]. In addition to these characteristics, they possess the uniform morphological structure, mechanical properties, and physical properties of synthetic fibers. Varying manufacturing techniques can be used to produce man-made regenerated fiber (rayon) thermoplastic composites, such as compression moulding, resin infusion [8,13], and injection moulding [10,14,15]. The flexural strength and modulus of rayon Elium^®^ composites obtained from the infusion technique were 93.5 MPa and 5.6 GPa, respectively [8]. The flexural modulus of rayon fiber PP composites after injection moulding was 3.88 GPa) [10].

Because of the lowest density and competitive price of polypropylene (PP) among other polymers, PP is known as a commonly used polymer [16]. PP is also chemical, biological, water-resistant, fatigue-resistant, and possesses insulating properties, and is recyclable. Therefore, the combination of PP and rayon fibers can offer a suitable fit for the requirements of lightweight applications. The construction of composites made from PP and rayon fibers can be altered to meet the needed requirements. Sandwich structures are one of the typical types of panels that provide a high bending stiffness-to-weight-ratio and are suitable to be used in key application areas such as marine, transport, sports, and leisure. An investigation [17] was carried out to study sandwich composites made from viscose fabrics and unsaturated polyester. Non-woven layers of viscose fabrics were used as the core material, while the warp knitted viscose fabrics were used as skin layers. Specimens were subjected to an incident energy of 25 J, and the structures with more warp knitted fabrics were found to reveal a greater impact force.

The aim of this project was to investigate and develop methods for recycling and reusing recycled paper machine textiles after their end-of-life as a component in a composite material. Due to its excellent mechanical properties, the textile could have a potential use in structural composites. These could be sandwich composites, in which the paper machine textile is used as the core material. The recycled textiles as core materials have the potential to withstand higher bending forces and induce greater stiffness than those of the bio-based composite materials. The inclusion of recycled textiles can not only improve the mechanical performance, but it can also make the composite part less expensive. To the best of the authors’ knowledge, very few reports [17] have studied the use of regenerated cellulose fibers in sandwich composites. Moreover, the utilisation of PP containing bio-based content to make sandwich composites has not been reported. Incorporation of recycled textiles in the regenerated cellulose fiber bio-based PP composite construction has not been introduced thus far. Therefore, in this work, the effects of the inclusion of various virgin/recycled polyamide (PA) textile fabrics into the rayon bio-based PP composite on the flexural, tensile, and low-velocity impact performance were investigated. The modes of failure of the sandwich specimens were also studied using an X-ray micro-computer tomography (µCT) image test with high-resolution images. The new class of sustainable sandwich composites investigated in this study can be utilised as lightweight composite structures suitable for various applications.

## 2. Materials and Methods

### 2.1. Materials

Regenerated cellulose fabrics (rayon) 0/90 were supplied by Cordenka company, Germany. The grade was 700 2440 dtex, with a density and area weight of 2482 dtex and 442 g/m^2^, respectively. The polymers used were bio-based polypropylene (PP) purchased from NaturePlast, France, and had 30 wt% bio content. The PP manufacturer provided the melt flow index (230 °C) and density of 70 g/10 min and 0.9 g/cm^3^, respectively. Paper machine textiles (felts) were provided by Albany International, Sweden. Felts were made of polyamide (PA) and supplied in both virgin and used forms. The textile acts as a substrate/support on which the pulp is formed into the paper product. These textiles are used under very demanding conditions, and they are highly engineered textile structures composed of yarns. Compaction of the felt, or loss of void volume, with time reduces the dewatering capacity of the felt, which is the main reason for its limited life or why felts are exchanged on a rather frequent basis. Moreover, the felt is subjected to various conditioning processes that wear the felt, resulting in fiber loss. Regarding the discolouration of felts after pulp and paper processing, it can be added that felts originally had a white colour. Discoloration relates to the process, or rather, the chemistry involved. The blue felt comes from a mill adding UV-light absorbents or whitening agents, which are most often blue. The brown roll is from another mill where pulp containing lignin is used. Before further processing, the used PA felts were washed thoroughly using lukewarm water for some minutes, rubbed simultaneously with hands to eliminate the impurities, and subsequently dried in a convection oven for 24 h at a temperature of 90 °C.

### 2.2. Processing

PP pellets were first used to make films of 0.25 mm thickness using a 20-tonne bench press machine (Rondol Industrie SAS, Nancy, France). The process conditions were optimised, as reported in our previous work [18]. To obtain the required thickness, 5 g of PP pellets were pre-heated at 200 °C between the top and bottom press plates for 1 min and subsequently pressed under the press area measuring 190 mm (length) × 190 mm (width) at 5 kN compressive force and the same temperature for another minute. The films were eventually cooled down for 3 min at room temperature and collected for composite manufacturing.

A film-stacking system was constructed by placing the alternative rayon fabrics, felt, and PP films to produce the sandwich composites with the aid of the same hydraulic press equipment that was used in the film-making process. A similar approach was carried out in our previous publication [18]. In fact, four thin PP film layers were utilised to bond the two layers of rayon fabrics to a PA felt (core) middle material, and therefore, the PA was the middle layer in the structure. The whole assembly was prepared for a dimension of 180 mm × 180 mm and then was pressed for 10 min at a pressure of 0.1 MPa and a temperature of 200 °C. Afterwards, the assembly was cooled down for 30 min at room temperature. It should be noted that the previously cut layers of rayon fabrics were dried in a convection oven for 24 h at 70 °C prior to the composite fabrications to eliminate the moisture content of the fibers.

Four different types of felt middle layers were used to form four distinct sandwich composites. The purpose was to examine the effect of varying thicknesses and recycled textiles obtained from different processes, which resulted in different types of contamination, on the mechanical behaviours of the sandwich composites. The use of waste textiles as a middle layer eliminates the probability of contamination since it is covered by top and bottom skins. In addition, they have porous structures that can act as core materials. When the virgin thick felt was used as the core layer, the composite was designated as VTC with a thickness of 5.0 mm. The composite with the virgin thin felt measuring 3.9 mm as the core layer was designated as VtC. The sandwich composites containing recycled felt materials were named brown core composite (BC) and blue core composite (BlC) and had the same thickness of 3.9 mm. The thin virgin felt and two used felts had a thickness of 2.6 mm, and the thickness of the thicker virgin felt was measured at 3.9 mm (Table 1).

In order to appropriately compare the results, in particular the bending behaviours, a control composite (C) possessing nearly the same weight as the sandwich structures containing the thinner cores was produced. Two layers of PP films were positioned between each of the two layers of fabrics (in total, four layers of fibers were incorporated), and on the very top and bottom layers of laminate, only one PP film was placed. The processing conditions, i.e., temperature, time, and pressure, of the composite laminate were identical to those carried out for the sandwich structures. The fiber mass fraction was determined to be approximately 50% for both the composite skin of the sandwich structures and the control composite. It is worth mentioning that the PP mass fraction in the whole sandwich composite containing the thinner cores is calculated at 27%. The composite plates were then cut into the required dimensions using a piece of laser cutting equipment (a GCC Laserpro Spirit GLS instrument) for different testing in accordance with the standards. Directly before the tests, the samples were conditioned for 24 h in a humidity chamber set to a temperature of 23 °C and a relative humidity of 50%.

### 2.3. Mechanical Properties and Damage Characterisations

The flexural tests were carried out on a Tinius Olsen H10KT universal testing instrument in accordance with the BS EN ISO 14125 standard [19]. For these three-point bending tests, the loadcell of 250 N was connected to the equipment, and the crosshead speed of 5 mm/min was used to apply the load to the samples measuring 80 mm (length) × 20 mm (width) in dimension. A span length of 64 mm was used. A total of five specimens were tested for each composite laminate, and the average values of flexural strength and modulus were reported for the composite plates, and the average values of bending force, initial slope of force-displacement curves, and displacements at maximum force were recorded for the sandwich composites.

The low velocity impact (LVI) experiments were carried out using the Instron CEAST 9340 drop weight machine. The experiments started by applying 15 Joules (J) incident energy to VTC. However, this amount of energy was not sufficient to damage or deform the sample. The incident impact energy was then increased from 15 J to 25 J, and this impact energy value was utilised to be applied to all the samples. The tests were carried out at room temperature, and all the tests were conducted in identical conditions. The LVI tests were conducted for five different types of materials, namely, VTC, VtC, BC, BlC, and control composite, and four specimens were tested for each material. The length and width of the LVI specimens were 70 mm × 70 mm. Force-displacement, force-time, and energy-time traces obtained from LVI tests were utilised to quantify and compare the impact response of these materials.

The tensile tests were performed with the same universal testing machine used for the flexural testing on all the composite laminates according to EN ISO 527-4 [20] (type 1B specimen). The dog bone samples were cut using the laser machine and were tested at a crosshead speed of 2 mm/min and a span length of 50 mm. A loadcell of 5 kN was used, and a 100R mechanical extensometer was connected to the middle of the samples possessing the initial distance between grips of 115 mm. The test was performed on five specimens of each composite, and the average values of tensile strength, modulus, and elongation at break were then reported.

An x-ray micro-computer tomography (µCT) image test was performed to investigate the failure of the test specimens after the LVI tests. A Nikon XTH 225 was used to scan the damage and the volume graphics VGSTUDIO MAX software (Version 2022.4) was used to render the 3D reconstructed volume. The micro-computer tomography (μ-CT) unit was set to an accelerating voltage of 135 kV and a beam current of 120 µA with no filtering and a molybdenum target metal. The unit is fitted with a Perkins Elmer flat panel detector. The resulting voxel size was 0.088 mm, and a total of 3600 images (with 0.1-degree intervals) were taken to produce the 3D reconstructed volume. The resulting images were processed on another computer to obtain the tomography images.

Digital imaging microscope tests were carried out to determine the bond strength between the inner core and the outer composite layer of the sandwich panels, as well as between the layers of fabric and the bio-based PP in the rayon composite laminate (control). A Nikon eclipse LV150 N microscope with a sophisticated optical system and digital imaging capabilities was used to examine the samples. Cross-sectional images of the samples were taken without any surface coating, enabling a thorough analysis of the bond quality at a microscopic scale.

## 3. Results

### 3.1. Flexural Properties of Composites

The bending tests were performed to study the effect of thicker, thinner felts as well as recycled felts as core materials on the bending force, displacement, and stiffness of the sandwich composites. The control composite was tested to provide a comparison in terms of flexural behaviours with those of the sandwich composites containing felts. The control and sandwich composites made of thinner cores were produced to offer the same weight for a reasonable evaluation of results (Table 2), except for the composite laminate consisting of thicker felt, which demonstrated a slightly higher weight. The ultimate force was found to be greatest for VTC, attributed to the higher thickness of the felt, which acts as a core material due to its porous structure. For this composite, an approximately 70% increment in the maximum force was obtained as compared to that of the control, which depicts the enhanced load-bearing capacity of the sandwich composite. The ultimate force was found to be lower than VTC for the sandwich composite specimens with thinner felt. Considering the variations in max. force for each composite, the sandwich composites containing thinner felts did not stand out from each other; however, they demonstrated higher values than those of the control.

The increased initial slope of the force displacement (Table 2) of the VTC composite specimens, which can be referred to as the greater stiffness, verified the effectiveness of the incorporation of the thicker core materials in the structure. If a ratio of 1 is given to the initial slope of the force vs. displacement for the control formulation, the stiffness displays 1.3 times more enhancement for BIC and BC sandwiches, 1.8 times more for VtC, and two times more for VTC composites than that of the control. In this case, the sandwich composites containing recycled cores (BLC and BC) presented a 0.3-time greater stiffness than that of VtC. This can be attributed to the compaction of the PA felts when they were initially used as a substrate in the formation of paper products. They lost their porosity and void volumes and also wore during paper-making processes under those demanding conditions. Displacement wise, the deflection at the maximum force was recorded as 30.2% and 28.8% greater for that of the VTC and sandwich composites with the thinner felt in comparison with that of the control, respectively. This is attributed to the ductility of the PA felts, which contributed to higher displacements, and the value is expected to be higher for the VTC as more PA existed in these specimens.

It was also revealed that all five different types of composites bent after the flexural tests and underwent a plastic deformation; however, no sign of delamination, visual microcracks, or debonding was detected. No indication of a dent in the samples through the flexural indenter was observed in the specimens as well.

### 3.2. Impact Damage Behaviour of Different Composites

**Figure 1:**
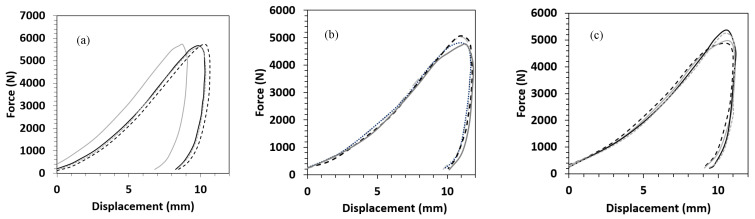
Force-displacment response of (**a**) Control, (**b**) VtC, and (**c**) BC after the impact drop tests on the composites.

**Figure 2:**
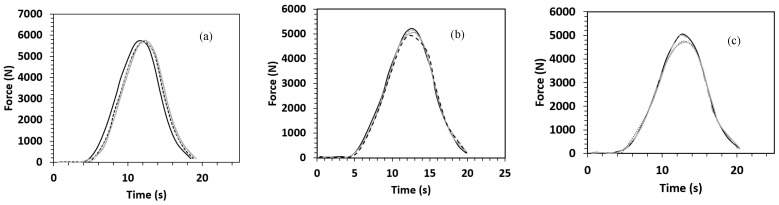
Force-time response of (**a**) Control, (**b**) BlC, and (**c**) VtC. after the impact drop tests on the composites.

**Figure 3:**
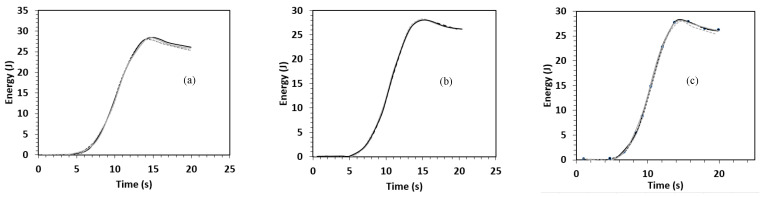
Energy-time response of (**a**) BlC, (**b**) VtC, and (**c**) BC after the impact drop tests on the composites.

**Figure 4:**
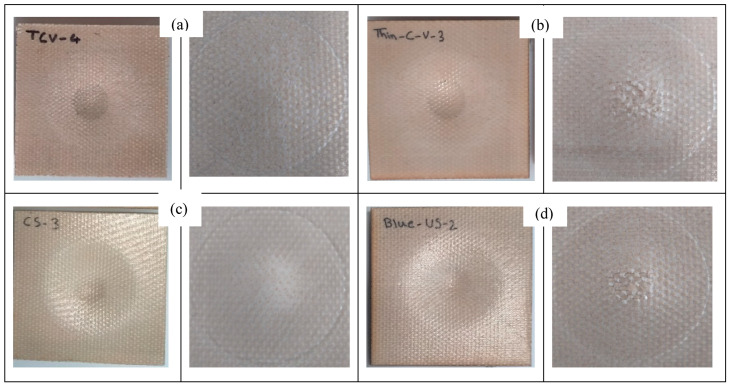

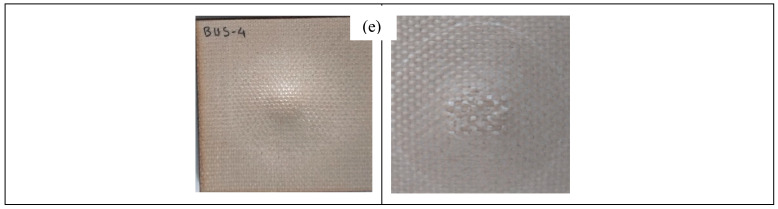
Visual inspection for perforation pattern of the front face of (**a**) the VTC, (**b**) VtC, (**c**) Control, (**d**) BlC, and (**e**) BC samples after the impact test (the images on the right side are the close-up versions).

The results for each material are presented in terms of force-displacement, force-time, and energy-time graphs and are depicted in Figure 1, Figure 2 and Figure 3. As seen in these graphs, the specimens in each material group demonstrate similar responses. This implies appropriate repeatability of the experiments. However, as shown in Figure 4, the control composite sample showed a slight variation compared to the others. Therefore, this sample was not used for making comparisons between different materials.

#### 3.2.1. Force-Displacement Response

**Figure 5:**
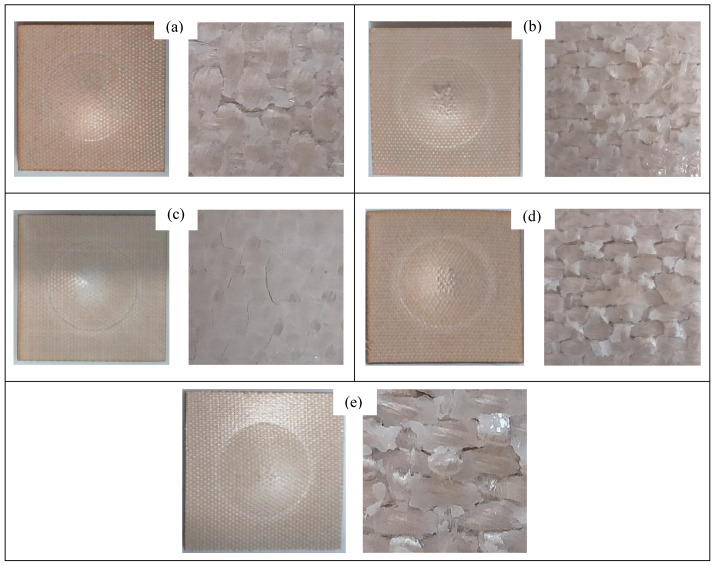
Visual inspection for perforation pattern of the rear side of (**a**) the VTC, (**b**) VtC, (**c**) control, (**d**) BlC, and (**e**) BC samples after the impact test (the images on the right side are the close-up versions).

Force-displacement curves after the drop test for three of the laminates are exhibited in Figure 1a–c, and the summary of the results is shown in Table 3. A very similar relationship can be observed between the force and displacement of all the composite laminates. The highest peak force was recorded at ca. 5229 N for VTC amongst all four sandwich composites, which is attributed to its thicker PA core, followed by BC, BlCC, and VtC laminates, which showed the maximum force in the range between approximately 4893 N and 5121 N. Due to the presence of more ductile rayon fabric layers in the control composite, the peak force was found to be at a higher value (5687 N). All the composites were tough, and the incident energy of 15 joules did not make a dent in the samples, and no sign of perforation was detected. Therefore, 25 J of incident energy was introduced to all the composites, and the damaged images of the samples under this impact energy are illustrated in Figure 4 and Figure 5. As seen in Figure 4 and Figure 5, it was observed that the specimens did not delaminate and the drop weight did not penetrate, indicating their failure resistance through energy absorption and dissipation; however, it was detected that the samples were dented in a circular shape on both sides. The force-displacement curves showed the stable propagation of the damage to the samples, and the maximum displacements of all the composite laminates were not very far from each other, falling within the range between 13.01 mm and 14.30 mm. It is promoted that the maximum displacements (Table 3) are in good agreement with the bending displacements at maximum force for the sandwich composite systems. Ude et al. [21] produced sandwich composites with silk-woven epoxy skins together with honeycomb (density = 0.056 kg/m^3^) as well as polymeric foam (0.052 kg/m^3^) as core material. The composite specimens were tested under an incident energy of 48 J, and it was revealed that sandwich composites with the honeycomb core and foam recorded peak forces of 800 N and 900 N, respectively. Another investigation [22] was performed on the sandwich composites with flax fiber epoxy composite skins and high-density polyethylene bottle caps as core materials. The peak force obtained from the drop test under an incident energy of 50 J was 1900 N. Hachemane et al. [23] made jute fiber epoxy composite skins and bonded them to cork agglomerate cores to produce sandwich composites. Under the impact energy of 16 J, the sandwich composite containing cork agglomerate cores with densities of 270 kg/m^3^ and 310 kg/m^3^ was found to demonstrate a peak force of 2700 N and 2000 N, respectively. It is worth mentioning that the peak forces obtained from the sandwich composite specimens with PA cores in this study presented higher values than the above-mentioned ones collected from the literature.

#### 3.2.2. Force-Time Behaviours

The force-time curves corresponding to the low velocity impact event for the composite specimens are presented in Figure 2a–c, and their respective parameters are shown in Table 3. The force is obtained as the reaction force exerted by the sample on the impactor tip. The force-time curves of all the composite specimens illustrated a mountain-like trend, nearly parabolic. In the case of thicker PA core material, the height of mountain-like curves improved as compared to other sandwich composites. Moreover, it was found out that the time to reach peak force was longer for this VTC sandwich composite than all other composites. The VtC, BC, and BlC sandwich composite specimens were found to reveal very close values of the time to peak force, which can be attributed to the same thickness of the sandwich composites in their structures. It is worth highlighting that the time taken to end the impact scenario was longer (t ≥ 20 ms) for all the sandwich composites than that of control (t ˂ 20 ms).

#### 3.2.3. Energy-Time Behaviours

The energy-time curves corresponding to the drop event for all the composites are depicted in Figure 3. At the incident energy of 25 J, the bio-based composite laminates were not punctured completely. This infers that the energy was absorbed and did not pass through the specimens. Moreover, it was figured out that all the sandwich composites and control demonstrated the same level of impact energy absorption, and the ultimate energy was recorded at about 28 J.

#### 3.2.4. Impact Damage and Failure Modes

The visual images taken from the post-impact tests are depicted in Figure 4 and Figure 5. Damage patterns on the top surfaces of the composite laminates showed clear matrix cracking (Figure 4), and the damage was more pronounced for the sandwich composites with the thinner core (VtC, BC, and BlC). However, VTC and Control were observed to experience less damage, as it is clearer in the close-up images.

The composite specimens detected did not show fiber pull-out, fiber breakage, and delamination. The damage propagation of the composite specimens was seen to be very similar. A circular-like effect was obvious on the front face of the specimens, where the spread of the impacted energy can be detected.

As seen in Figure 5 (close-up views), matrix cracking and damage propagation were more conspicuous in the sandwich composites containing thinner PA cores (VtC, BC, and BlC). These sandwich composite specimens were found visually to be very similar in their failure patterns owing to their similar composite configurations. In the case of VTC, less deformation and fewer cracks were observed upon impact, implying that the sandwich composite with a thicker core was more impact resistant due to the greater absorption of the energy dissipation.

**Figure 6:**
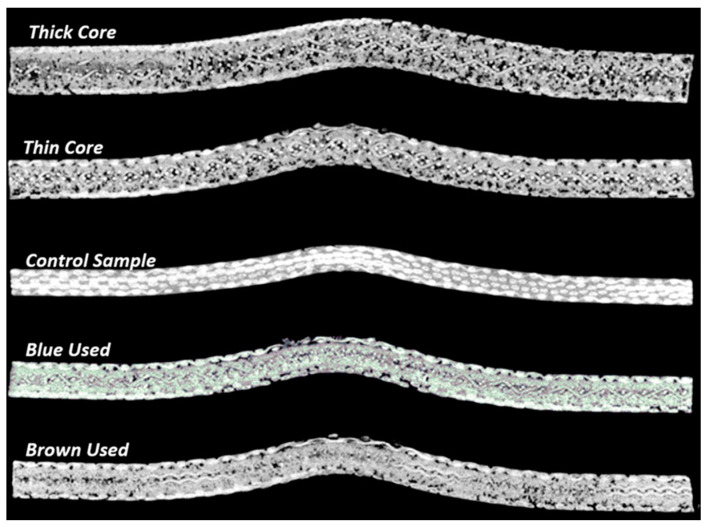
CT images of the composite laminate specimens in impact zone cross-section.

The X-ray micro-CT images of the specimens after the impact tests are illustrated in Figure 6. The deformation mechanisms of the composite samples in the area of cross-section are illustrated in the CT images. No fiber bundle breakage, tow debonding, fiber bundle splitting, or delamination could be found in the 2D cross-section slices of the composite specimens. Moreover, no obvious cracks were observed directly underneath the contact zone. In other studies, the X-ray micro-CT tests were performed on carbon-epoxy-based composites [24,25], and similar modes of failure were observed for the plain carbon fabric-reinforced epoxy composite specimens at an impact energy of 35 J [24].

### 3.3. Tensile Test Analysis of the Composites

The tensile strength and modulus of the composite laminates are shown in Table 4. The control specimen was found to demonstrate tensile strength and modulus of 91.90 MPa and 1.35 GPa, respectively. When the sandwich composite samples were pulled from the ends, it was detected that the tensile strength decreased to a value range of approximately 34–46 MPa. Similarly, the tensile modulus was seen to drop to a lower value of 0.36–0.67 GPa as compared to that of control. The reasoning behind these is that more (rayon) fibers existed in the control composite, which led to higher properties of the composite in the tension mode, given the fact that the tensile test is a fiber-dependent characterisation. Moreover, since the sandwich structures possessed a cellular-like structure with numerous amounts of porosity, a direct calculation of tensile strength and modulus is expected to be lower as compared to that of the control. Furthermore, the presence of porous core material, as expected, reduced the tensile modulus as the through thickness was not fully covered by the PA material. Moreover, PA has a lower modulus than rayon fibers, as the current grade of rayon fiber has a 14.3 GPa tensile modulus. VTC was seen to have a lower tensile modulus among other composites, attributed to its thicker PA core material.

## 4. Discussion

The aim was to utilise both virgin and used textile fabrics due to their porous structures as a central element in the composites. Additionally, the textile was placed between two layers of facings, eliminating any potential contamination concerns.

Therefore, instead of simply discarding the materials by throwing them into the landfill, we have the opportunity to utilise them more effectively by incorporating them as reinforcement in the body of composites.

Compared to typical standard sandwich structures that utilise standard cores such as foams and balsa wood [18], it could be argued that the highest bending properties are not expected to be achieved with these types of constructed panels. However, these panels can still offer a good level of bending properties, particularly for less demanding applications such as semi-structural parts.

For the drop weight (impact) test, it is important to note that the maximum forces obtained from the sandwich composite specimens with PA cores in this research showed higher values compared to the previously mentioned ones found in the literature from the typical sandwich panels (the values obtained from the literature are compared with the findings from the current work in Section 3.2.1).

**Figure 7:**
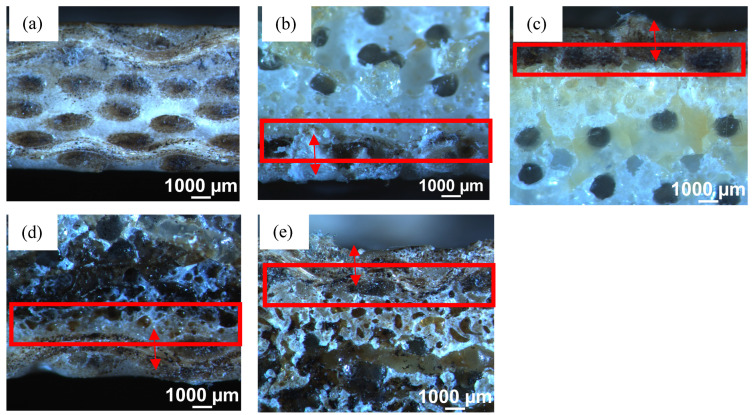
Microscopy images of (**a**) Control, (**b**) VTC, (**c**) VtC, (**d**) BlC, and (**e**) BC.

To evaluate the bonding between the skins and the textile material, it is recommended to observe the interface between the layers using microscope equipment. The microscope images of the control and sandwich composite specimens are shown in Figure 7. As can be seen in all the microscope images of the composites taken under the microscope, the bonding between the layers of the control specimen was excellent (Figure 7a), and is successful in all formulations. Figure 7b,c show the microscope pictures of VTC and VtC, respectively. The interface of these two sandwiches exhibits the virgin textile core, which is depicted as white in colour, and a layer of rayon fabric, which was successfully impregnated together. The interface between the layers is displayed in a red rectangle, and the thickness of the facing is presented by a red arrow. Similarly, the interface between the textile core and the rayon composite skin is depicted in Figure 7d,e, for the sandwich composites containing the end-of-life textiles. The bonding for BlC and BC composites was found to be excellent.

## 5. Conclusions

The sandwich composites containing regenerated cellulose fabric, bio-based PP composite skins, and recycled PA textiles were produced successfully using the hot press method. The aim was to utilise end-of-life textiles from industry and provide a secondary application for them. It was discovered that substituting these recycled textiles for rayon fabrics can maintain the mechanical performance of the composite laminate. In particular, due to the porosity and durability of these PA textiles, greater bending force and stiffness than those of control were achieved. For the LVI tests, the sandwich composites were found to reveal comparable LVI energy (ca. 28 J), maximum displacement (~13–14 mm), and peak force (in the range of 4.8–5.2 kN) with those of the control. A good agreement between the bending and impact test results in terms of displacement was observed. Force-wise, nearly the same trend was found for different sandwich composites (VTC > VtC, BC, BlC) as obtained from the flexural and drop impact tests. Tensile strength and modulus of sandwich composites were lower than those of control due to the porous structure of the textiles used as core materials and also the presence of more rayon fibers, which possess higher tensile properties than PA core, in the control composite. As observed in the visual inspection of the sandwich laminates, bio-based sandwich composite specimens containing the recycled cores showed larger microcracks than VTC and control. The CT images illustrated no fiber delamination, bundle breakage, or fiber bundle splitting after the drop test. These digital and CT images evidenced good bonding between PA cores and bio-based PP composite skins. All the results obtained prove the potential of end-of-life textile materials for use in secondary applications, especially-composite associated components. This type of usage can lower the costs of composite fabrication while simultaneously providing sufficient mechanical properties. It can be concluded that the inclusion of used textiles can address both sustainable issues and cost-property benefits and eventually lead to low-cost, eco-friendly, and lightweight composite structures. It is interesting to investigate the effect of using end-of-life or waste textiles as reinforcement in natural fiber thermoset systems. Therefore, our future work will focus on studying these types of composites. Additionally, we will also examine other types of textile materials.

## Figures and Tables

**Table 1:** Composite formulations.

Sample	Impurities of the Textile Core before Cleaning	Type of Felt	Thickness of Felt (mm)	Thickness (mm)
Composite with a virgin thick core (VTC)	No contamination	virgin	4	5
Composite with a virgin thin core (VtC)	No contamination	virgin	2.6	3.9
Composite with a blue core (BlC)	UV light absorbents or whitening agents	used	2.6	3.9
Composite with a brown core (BC)	Pulp containing lignin	used	2.6	3.9
Control (C)	NA	not used	2.6	3.9

**Table 2:** The flexural properties of the control and sandwich composites.

Sample	VTC	VtC	BlC	BC	C
Thickness (mm)	5.0	3.9	3.9	3.9	2.8
Weight ratio	~1.1	~1	~1	~1	1
Max Force (N) [SD]	84.4 [1.88]	71.2 [5.4]	75.3 [6.7]	67.3 [2.1]	49.69 [3.3]
Average initial slope (of force-displacement curves)	33.3	30.0	21.4	21.6	16.7
initial slope ratio	~2.0	~1.8	~1.3	~1.3	1.0
Average displacement (mm) [SD] at max. force	14.9 [0.2]	13.4 [0.8]	13.4 [1.0]	13.4 [0.3]	10.4 [0.8]

**Table 3:** Average drop weight impact test results for the control and sandwich composites.

Specimens	Average Maximum Energy (J)	SD (J)	Average Maximum Force (N)	SD (N)	Max. Displacement (mm)	SD (mm)
Control	28.13	0.02	5687.83	47.21	13.01	0.18
VtC	28.32	0.014	4893.21	158.07	14.30	0.09
BC	28.16	0.14	5121.07	224.47	13.70	0.09
VTC	28.13	0.27	5229.23	69.34	13.96	0.56
BlC	28.06	0.19	5061.94	102.16	13.79	0.21

**Table 4:** Tensile strength and modulus of the sandwich composites and Control.

Materials	Strength (MPa)	SD (Strength)	Tensile Modulus (GPa)	SD (Tensile Modulus)
Control (C)	91.90	0.80	1.35	0.05
Virgin thin core composite (VtC)	34.20	1.80	0.56	0.01
Brown core composite (BC)	45.70	1.80	0.49	0.01
Virgin thick core composite (VTC)	36.00	1.40	0.36	0.01
Blue core composite (BlC)	46.80	2.60	0.67	0.01

## Data Availability

Data are available upon request.
